# Osteoid osteoma of the ethmoid bone associated with dacryocystitis

**DOI:** 10.1186/1746-160X-2-23

**Published:** 2006-08-04

**Authors:** Vassilios A Lachanas, Anastasios V Koutsopoulos, Jiannis K Hajiioannou, Argyto J Bizaki, Emmanuel S Helidonis, John G Bizakis

**Affiliations:** 1 Department of Otolaryngology, University of Crete School of Medicine, Heraklion, Crete, Greece; 2 Department of Pathology, University of Crete School of Medicine, Heraklion, Crete, Greece

## Abstract

**Background:**

Osteoid osteomas (OO) are small, benign osteoblastic lesions. Ethmoid bone OO has been very rarely reported so far.

**Case presentation:**

We report a case of a 16-year-old boy suffering from persistent epiphora and a mild pain in the area of median canthus, due to a bone density mass within the right ethmoid air cells extending to the ipsilateral right orbit. The mass was removed via an external ethmoidectomy approach. Histopathologic examination of the specimen set the diagnosis of OO. One year after the operation the patient is free of symptoms, while no recurrence occurred.

**Conclusion:**

A case of ethmoid bone OO associated with dacryocystitis is reported. Although benign and rare, OO should be considered in differential diagnosis of the ethmoid bone osteoblastic lesions.

## Background

Osteoid osteoma was firstly described by Jaffe in 1935. It is a small, benign osteoblastic tumor, comprising 12% of the benign osseous tumors. Osteoid osteoma is characterized by varying intermixtures of osteoid, newly formed bone, and highly vascular supporting osseous tissue (nidus) surrounded by a distinctive surrounding zone of reactive bone formation. The nidus typically measures less than 1.5 cm in diameter [1].

Osteoid osteoma most commonly (75%) occurs between 5 and 25 years of ages, with the majority of cases being the 2^nd ^decade of life, while it is distinctively rare above the age of 30. There is a male predominance, with a 2–3:1 male to female ratio. Osteoid osteomas occur most commonly in the femur (27.33%), tibia (22.1%) and spine (10%) [1-3]. The less frequent sites of involvement are the ribs, the mandible and the calvarium [1].

We report a very rare case of an ethmoid bone osteoid osteoma associated with dacryocystitis. The clinical, radiological, and pathological features are addressed.

## Case presentation

A 16-year-old boy was referred to our department by his ophthalmologist. He was suffering from persistent epiphora for about one year, which had been diagnosed as chronic dacryocystitis due to right nasolacrimal duct obstruction, while he had undergone probing of the right nasolacrimal duct once, by his ophthalmologist. The patient also referred a mild pain in the area of median canthus, which was more intense during the night and relieved with aspirin uptake. In our department, clinical examination revealed only a small, firm mass, palpated in the middle angle of the right orbit. Nasal endoscopy revealed no intranasal pathology. There was normal ocular motility and no eyeball displacement.

**Figure 1:**
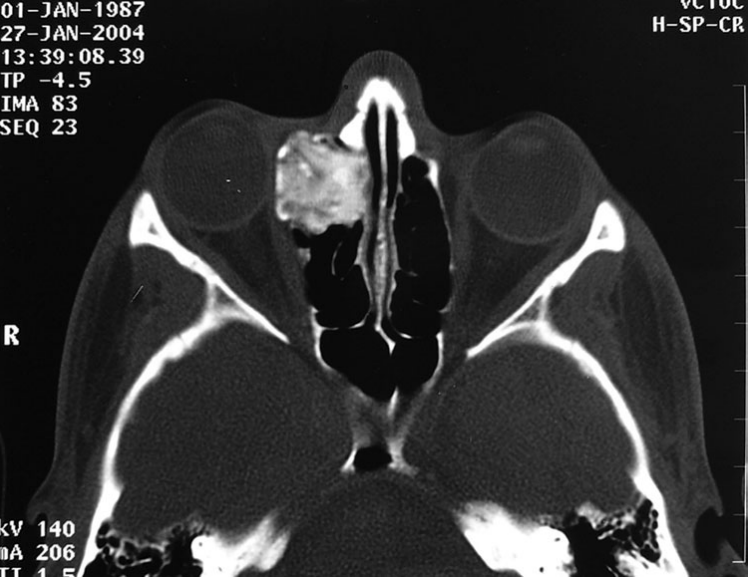
**Preoperative CT scan**. CT scan of the paranasalsinuses, demonstrating a bone density mass within the right ethmoidair cells, with well defined margins, extending laterally through thelamina papyracea, to the ipsilateral right orbit.

**Figure 2:**
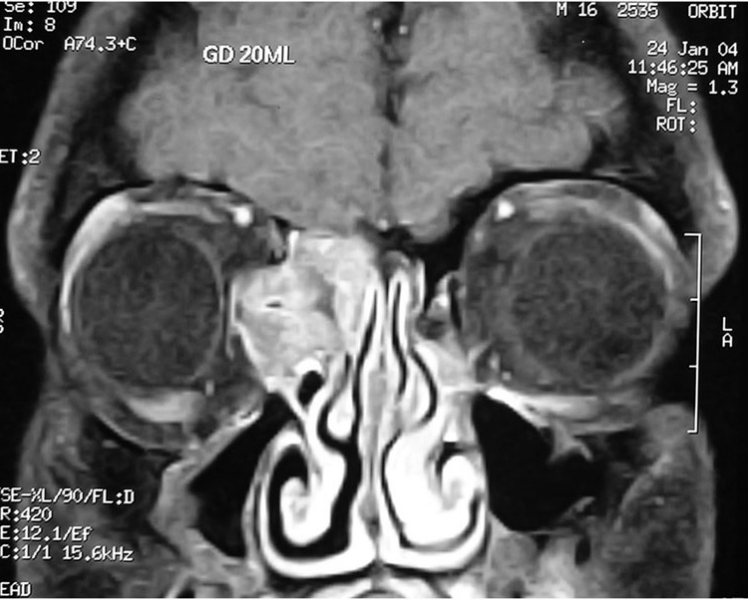
**Preoperative MRI scan**. Coronal T_1_W post-contrast image MRI scan, showing a mass filling the anterior portion of the right ethmoid air cells. The mass demonstrates patchy enhancement, protrudes into the adjacent orbit, depicts soft tissue intensity, and enhances following contrast administration.

A Computed Tomography scan was performed, which showed a bone density mass within the right ethmoid air cells. The mass revealed sharp and well defined margins, extending laterally through the lamina papyracea, to the ipsilateral right orbit (Figure 1). Subsequently, a Magnetic Resonance Imaging (MRI) scan was performed. Coronal, T1-weithted, post-contrast image, confirmed the presence of the mass filling the anterior portion of the right ethmoid air cells. The mass demonstrated patchy enhancement and protruded into the adjacent orbit. It should be noted that on MRI the mass depicted soft tissue intensity and enhanced following contrast administration (Figure 2).

**Figure 3:**
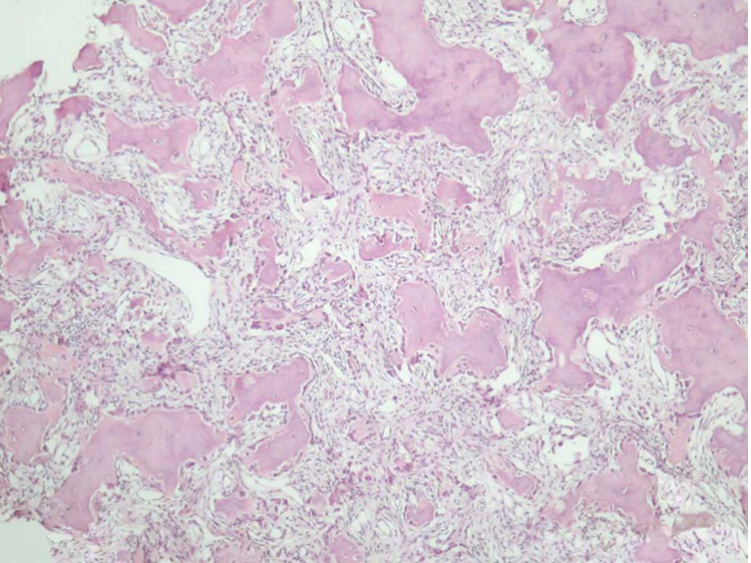
**Microphotograph of the tumor**. Varying intermixtures of osteoid, newly formed bone in a highly vascular supporting fibrous tissue (Nidus). (H & E stain – original magnification ×40).

**Figure 4:**
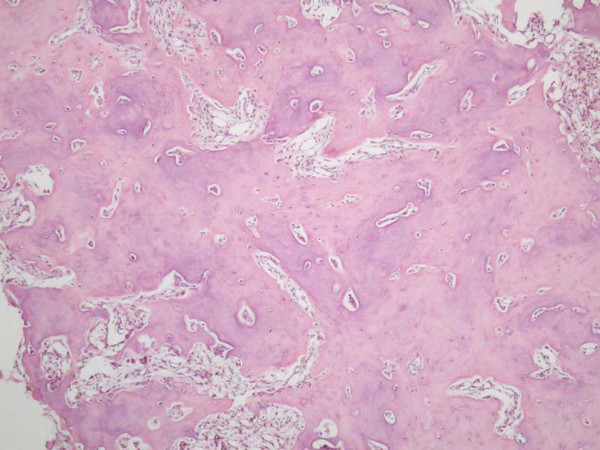
**Microphotograph of the tumor**. Dense sclerotic bone formation surrounding the nidus. (H & E stain – original magnification ×40).

The mass was removed via an external ethmoidectomy approach. Intraoperatively it was noticed that the mass compressed the lacrimal sac, while no dacryocystorhinostomy was performed. Histopathological examination revealed that, the specimen mostly consisted of cancellous bone particles, some of which appeared to have distorted architecture, because of an interlacing network of variably sized, shaped and mineralized osteoid trabeculae. There was a rimming of osteoblasts surrounding the trabeculae. The intertrabecular spaces were occupied by loose connective tissue (Figure 3). Focally dense sclerotic bone was recognized surrounding the above structures (Figure 4). Diagnosis of ethmoid bone Osteoid Osteoma was set.

One year after the operation the patient was free of symptoms, while no recurrence occurred.

## Discussion

Osteoid osteomas are small, benign osteoblastic tumors. The lesion tends to involve the cortex rather than the medulla, and has limited growth potential. Osteoid osteomas have been reported almost in every bone of the human body, while the majority is met in metaphysis or shaft of long bones [2]. Most commonly occurs in the femur, tibia and spine, while the ribs, the mandible and the calvarium are the rarest sites of involvement [1,2]. Grayeli et al reported a case of posterior ethmoid osteoid osteoma [4], Banerjee et al reported a case of Osteoid osteoma of the ethmoid associated with pneumocephalus [5], while Pai et al reported a large osteoid osteoma of the ethmoid with intraorbital and intracranial extension [6]. To our knowledge (Medline search) this is the forth case of ethmoid osteoid osteoma in English literature, while association with dacryocystitis has never been reported so far.

Clinical symptoms depend on the location of the lesion. The most notable of the early symptoms is an intermittent vague pain, gradually increasing in severity, with nocturnal paroxysm. This pain responds characteristically to aspirin treatment [1-3,7]. In our case, the patient suffered from dacryocystitis, due to compression of the lacrimal sac from the osteoid osteoma. Surgical removal of the mass was sufficient in relieving patient's symptoms, while no further intervention in the lacrimal apparatus was performed. Since dacryocystitis might, even rarely, be due to intranasal pathology, we believe that otolaryngologic evaluation should be performed in patients with persistent symptoms.

Computed tomography is the imaging modality of choice to detect osteoid osteoma, demonstrating a small osteolytic lesion less than 1.5 cm in diameter with a dense sclerotic ring, which has in some cases (20–30%) central calcifications. It should be noted that osteoid osteoma might be even completely calcified [8]. Magnetic Resonance Imaging appearance depends on the amount of calcification within the nidus, the size of the fibrovascular zone, reactive sclerosis and the amount of edema in the bone; so it may not be diagnostic [9].

Macroscopically osteoid osteoma nidus can be recognized mostly within the cortex and less frequently in the spongiosa. Its configuration varies from oval to globular, with clear and distinct delimitation from the adjacent osseous tissue. The lesion is usually brownish-red and mottled with granular gritty consistency. The histologic hallmark of osteoid osteoma is the nidus, which is characterized by varying intermixtures of osteoid, newly formed bone and highly vascular supporting fibrous tissue. The osteoid may appear in broad sheets in some areas, or it may present bony trabeculae in the process of calcification or ossification. The trabeculae are thin and show prominent osteoblastic rimming. The nidus is surrounded by thickened cortical bone [1,2].

Clinical differential diagnosis of osteoid osteoma includes osteomyelitis and osteoblastoma. Osteomyelitis may form a localized abscess termed "Brodie's abscess", which on roentgenogram can simulate the appearance of osteoid osteoma. Histopathologicaly, however, Brodie's abscess shows inflammation and not a bony nidus [2]. Osteoblastoma has inconsistent pain, rapid increase in size, and the lesion usually measures more than 2 cm [1]. On histopathologic examination the great difficulty is to distinguish osteoid osteoma from osteoblastoma, because of the similarity of histopathological features. In these cases, diagnosis is based on the size of the nidus, and the presence of reactive bone formation, while active osteoblasts are more numerous, the stroma is richly vascularized and extravasated blood with large number of multinucleated giant cell macrophages are noted [1,2,6]. In practice a lesion smaller than 1.5 cm is considered as osteoid osteoma and a lesion larger than 1.5 cm as osteoblastoma [1,2]. In our case histopathologic diagnosis was based on the size of the lesion, which was less than 1.5 cm (1.5 × 1 × 0.4 cm), as well as on the presence of reactive dense sclerotic bone formation.

## Conclusion

In conclusion, by presenting this case we would like to report the forth case of ethmoid osteoid osteoma in English literature, which should be considered in differential diagnosis of the osteoblastic lesions of the ethmoid bone. Furthermore, we believe that since dacryocystitis might, even rarely, be due to intranasal pathology, otolaryngologic evaluation should be performed in patients with persistent symptoms.

## Competing interests

The author(s) declare that they have no competing interests.

## Authors' contributions

**VL: **participated in the surgical procedure and drafted the manuscript. **AK: **did the histopathological examination, conceived of the report, and helped in drafting the manuscript. **JH: **participated in the surgical procedure, in bibliographical data collection and helped to draft the manuscript. **AB: **participated in bibliographical data collection and helped to draft the manuscript. **EH: **helped to draft the manuscript, and helped to the critical review of the manuscript. **JB: **performed the surgical procedure, helped to draft the manuscript, and helped to the critical review of the manuscript. All authors read and approved the final manuscript.

## References

[B1] Huvos GA (1991). Osteoid Osteoma. Bone tumors, diagnosis, treatment and prognosis.

[B2] Unni KK, Inwards CY, Christofer DM Fletcher (2002). Tumors of the osteoarticular system. Diagnostic Histopathology of Tumors.

[B3] Dahlin DC, Unni KK, Charles CT (1987). Bone tumors. General aspects and data on 8542 cases.

[B4] Grayeli AB, Redondo A, Sterkers O (1998). Anterior skull base osteoid osteoma: case report. Br J Neurosurg.

[B5] Banerjee T, Meagher JN, Donley C (1975). Osteoid osteoma of the ethmoid and pneumocephalus. South Med J.

[B6] Pai BS, Harish K, Venkatesh MS, Shankar U, Deepthi J (2005). Ethmoidal osteoid osteoma with orbital and intracranial extension. BMC Ear Nose Throat Disord.

[B7] Greenspan A (1993). Benign bone-forming lesions: osteoma, osteoid osteoma, and osteoblastoma. Clinical, imaging, pathologic, and differential considerations. Skeletal Radiol.

[B8] Bahloul K, Xhumari A, Feydy A, Kalamarides M, Redondo A, Rey A (2003). Thoracic spine osteoid osteoma. European Journal of Radiology Extra.

[B9] Hermann G, Abdelwaheb F, Casden A, Mosesson R, Klein MJ (1999). Osteoid osteoma of a cervical vertebral body. Br J Radiol.

